# Motor cortex neurons: from experiment to model via evolutionary algorithms

**DOI:** 10.1186/1471-2202-16-S1-P152

**Published:** 2015-12-18

**Authors:** Samuel A Neymotin, Benjamin A Suter, Michele Migliore, Salvador Dura-Bernal, Gordon MG Shepherd, William W Lytton

**Affiliations:** 1 Department Physiology & Pharmacology, SUNY Downstate, Brooklyn, NY, 11203, USA; 2 Department Physiology, Northwestern University, Chicago, IL, 60611, USA; 3 Institute of Biophysics, National Research Council, Palermo, Italy; 4 Department of Neurology, Kings County Hospital Center, Brooklyn, NY, 11203, USA

## 

There are several types of neurons in primary motor cortex, with differences in ion channels, dynamics, morphology, as well as differences in synaptic input localization and axonal projections. To constrain computer models of cortical neurons, it is necessary to record from these different types of neurons. Model parameters can then be optimized, to minimize the difference between the model and experimental data.

We have performed experiments recording from 2 classes of layer 5 pyramidal neurons in mouse motor cortex: corticospinal (SPI) and corticostriatal (STR). These neurons exhibit differences in synaptic inputs, axonal projections, and intrinsic membrane properties. Somatic whole-cell recordings were performed in both classes and current steps were injected (-0.2 to 0.6 nanoamperes) to produce different dynamical behaviors, including hyperpolarization-induced sag, alterations in firing rate and spike timing. We have developed multiple computer models of the different neuronal classes, each with varying complexity, and all constrained by experimental data. Two optimization methods were used: 1. evolutionary algorithms, 2. NEURON's principal axis (praxis) error minimization algorithm. We optimized model parameters with multiple fitness functions, first independently, and then in different combinations. The fitness functions quantified differences between experimental and simulated data, for firing frequency, resting membrane potential (RMP), action potential shape, interspike interval voltage trajectory, spike timing, hyperpolarization-induced *sag*, and voltage responses to onset/offset of current injections.

We assessed optimized performance of different classes of neuronal compartmental models, with varying levels of morphological complexity, across the fitness functions. The models included a 5-compartment pyramidal neuron (with soma, basal dendrite, and three apical dendritic segments), and a ~904-compartment pyramidal neuron with detailed dendritic and axonal geometry reconstructed from our experiments. All model neurons included the same set of ion channels: INa and IKdr for action potential generation; IKa for rapid repolarization following an action potential; IKm and IKd for afterhyperpolarization; Ih for resonance, sag, excitability, and contribution to RMP; calcium channels (L, N, T-type) for bursting, excitability modulation, and contribution to backpropagating action potentials; calcium-activated potassium channels for regulating excitability after depolarization-induced calcium influx.

We performed optimization in two phases: 1. optimize passive properties and compartmental geometry; 2. optimize maximal conductance and kinetics of each ion channel. The distribution of ion channels were constrained by patterns from experimental literature. For example, Ih increased exponentially with distance from the soma, while somatic and axonal compartments had higher densities of Na and K channels to allow action potential generation. This also reduced free parameters for optimization. Across the different models, the firing frequency, sag, and RMP had nearly perfect fits to the experimental data, while using a spike timing fitness function reduced spike time errors to within 1-10 ms. Due to the high dimensionality of parameter space, different subsets of fitness functions tended to improve independently, while others were correlated. We assessed which parameter values and ion channels had the largest impact on each fitness function using a post-optimization sensitivity analysis. Our methods were effective in optimizing both SPI and STR neurons and will likely generalize to other cell types.

